# Disease severity prognostication in primary sclerosing cholangitis: a validation of the Anali scores and comparison with the potential functional stricture

**DOI:** 10.1007/s00330-024-10787-4

**Published:** 2024-06-13

**Authors:** Sarah Poetter-Lang, Ahmed Ba-Ssalamah, Alina Messner, Nina Bastati, Raphael Ambros, Antonia Kristic, Jakob Kittinger, Svitlana Pochepnia, Sami A. Ba-Ssalamah, Jacqueline. C. Hodge, Emina Halilbasic, Sudhakar K. Venkatesh, Nikolaos Kartalis, Kristina Ringe, Lionel Arrivé, Michael Trauner

**Affiliations:** 1 Department of Biomedical Imaging and Image-guided Therapy, Medical University Vienna, Vienna, Austria; 2 Division of Gastroenterology and Hepatology, Department of Internal Medicine III, Medical University of Vienna, Vienna, Austria; 3 Department of Abdominal Imaging, Department of Radiology, Mayo Clinic, Rochester, MN USA; 4 Division of Radiology, Department of Clinical Science, Intervention and Technology (CLINTEC), Karolinska Institute, Stockholm, Sweden; 5 Department of Diagnostic and Interventional Radiology, Hannover Medical School, Hannover, Germany; 6 Department of Radiology, Saint-Antoine Hospital, Assistance Publique - Hôpitaux de Paris (APHP) and Sorbonne University, Paris, France

**Keywords:** Cholangitis (sclerosing), Constriction (pathologic), Magnetic resonance imaging (functional), Contrast media, Cholangiopancreatography (prognosis)

## Abstract

**Objectives:**

Our aim was twofold. First, to validate Anali scores with and without gadolinium (ANALI_Gd_ and ANALI_NoGd_) in primary sclerosing cholangitis (PSC) patients. Second, to compare the ANALIs prognostic ability with the recently-proposed potential functional stricture (PFS).

**Materials and methods:**

This retrospective study included 123 patients with a mean age of 41.5 years, who underwent gadoxetic acid-enahnced MRI (GA-MRI). Five readers independently evaluated all images for calculation of ANALI_Gd_ and ANALI_NoGd_ scores based upon following criteria: intrahepatic bile duct change severity, hepatic dysmorphia, liver parenchymal heterogeneity, and portal hypertension. In addition, hepatobiliary contrast excretion into first-order bile ducts was evaluated on 20-minute hepatobiliary-phase (HBP) images to assess PFS. Inter- and intrareader agreement were calculated (Fleiss´and Cohen kappas). Kaplan-Meier curves were generated for survival analysis. ANALI_NoGd_, ANALI_Gd_, and PFS were correlated with clinical scores, labs and outcomes (Cox regression analysis).

**Results:**

Inter-reader agreement was almost perfect (ϰ = 0.81) for PFS, but only moderate-(ϰ = 0.55) for binary ANALI_NoGd_. For binary ANALI_Gd_, the agreement was slightly better on HBP (ϰ = 0.64) than on arterial-phase (AP) (ϰ = 0.53). Univariate Cox regression showed that outcomes for decompensated cirrhosis, orthotopic liver transplantation or death significantly correlated with PFS (HR (hazard ratio) = 3.15, *p* < 0.001), ANALI_NoGd_ (HR = 6.42, *p* < 0.001), ANALI_Gd_HBP (HR = 3.66, *p* < 0.001) and ANALI_Gd_AP (HR = 3.79, *p* < 0.001). Multivariate analysis identified the PFS, all three ANALI scores, and Revised Mayo Risk Score as independent risk factors for outcomes (HR 3.12, *p* < 0.001; 6.12, *p* < 0.001; 3.56, *p* < 0.001;3.59, *p* < 0.001; and 4.13, *p* < 0.001, respectively).

**Conclusion:**

ANALI_NoGd_ and GA-MRI-derived ANALI scores and PFS could noninvasively predict outcomes in PSC patients.

**Clinical relevance statement:**

The combined use of Anali scores and the potential functional stricture (PFS), both derived from unenhanced-, and gadoxetic acid enhanced-MRI, could be applied as a diagnostic and prognostic imaging surrogate for counselling and monitoring primary sclerosing cholangitis patients.

**Key Points:**

- *Primary sclerosing cholangitis patients require radiological monitoring to assess disease stability and for the presence and type of complications*.
- *A contrast-enhanced MRI algorithm based on potential functional stricture and ANALI scores risk-stratified these patients*.
- *Unenhanced ANALI score had a high negative predictive value, indicating some primary sclerosing cholangitis patients can undergo non-contrast MRI surveillance*.

**Graphical Abstract:**

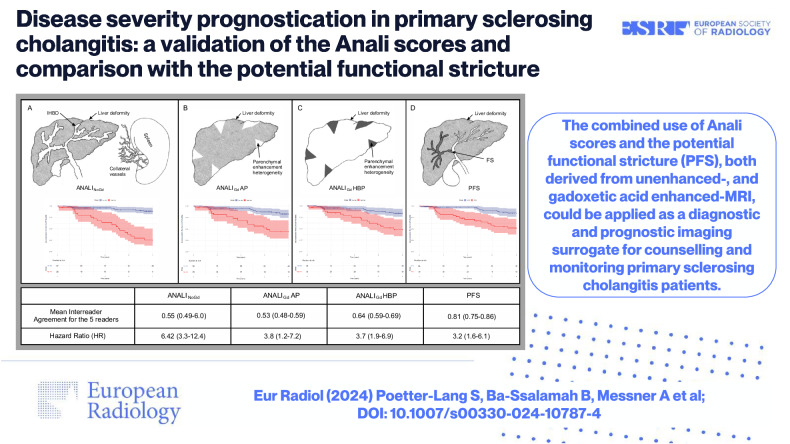

## Introduction

Primary sclerosing cholangitis (PSC) is a low-prevalence chronic progressive inflammatory disease [1]. Although elevated cholestatic laboratories are suggestive, they can neither diagnose nor monitor PSC activity over its median 12–20-year duration [2–4]. Therefore, an alternative non-invasive exam is needed to diagnose PSC, detect its complications, including the development of clinically-relevant strictures, and predict outcomes. Meta-analysis, confirming international imaging guidelines, showed that non-invasive magnetic resonance cholangiopancreatography (T2-MRCP) is the imaging study of choice for diagnosing PSC due to its high accuracy and very high (94%) specificity and positive likelihood ratio [5].

Ruiz et al developed the Anali scoring system with and without extracellular Gd-chelate to predict the most important factors determining PSC survival, including liver decompensation, liver-related death and/or the need for orthotopic liver transplant (OLT) [6]. Lemoinne et al validated both scores using an external cohort [7]. The Anali scores accurately predicted 4-year radiologic progression from baseline with subsequent validation in a retrospective multi-center study [6, 7]. Grave et al confirmed moderate inter-reader agreement for the Anali no gadolinium score and Anali arterial-phase using extracellular gadolinium-based contrast agent, as well as their predictive value [8]. Although, Grigoriadis et al found poor to moderate inter-reader agreement for the Anali no gadolinium score and Anali arterial-phase with gadoxetic acid, challenging their clinical utility, they also confirmed that both Anali scores correlated with clinical outcomes, highlighting MRI’s value in determining PSC prognosis [9].

Recently, Poetter-Lang et al reported on the value of gadoxetic acid-enhanced MRI (GA- MRI) to non-invasively and confidently diagnose potential functional stricture (PFS) in PSC patients, with an additional predictive value [10]. They found that this simple binary stratification could not only diagnose FS requiring ERCP dilatation and/or stenting, but also predict OLT or liver-related death in their cohort by diagnosing very advanced-stage PSC, i.e., hepatocellular dysfunction (HD) [10].

In PSC patients, both the Anali scores and PFS can be simultaneously evaluated prognostically on GA-MRI, including T2-weighted MRCP. Therefore, using gadoxetic acid, instead of conventional gadolinium chelates, our purpose was two-fold, firstly, to validate Anali scores with and without gadolinium (ANALI_Gd_AP, ANALI_Gd_HBP and ANALI_NoGd_) and, secondly, to compare their prognostic ability with the recently-proposed potential functional stricture (PFS).

## Patients and methods

### Patients

**Fig. 1:**
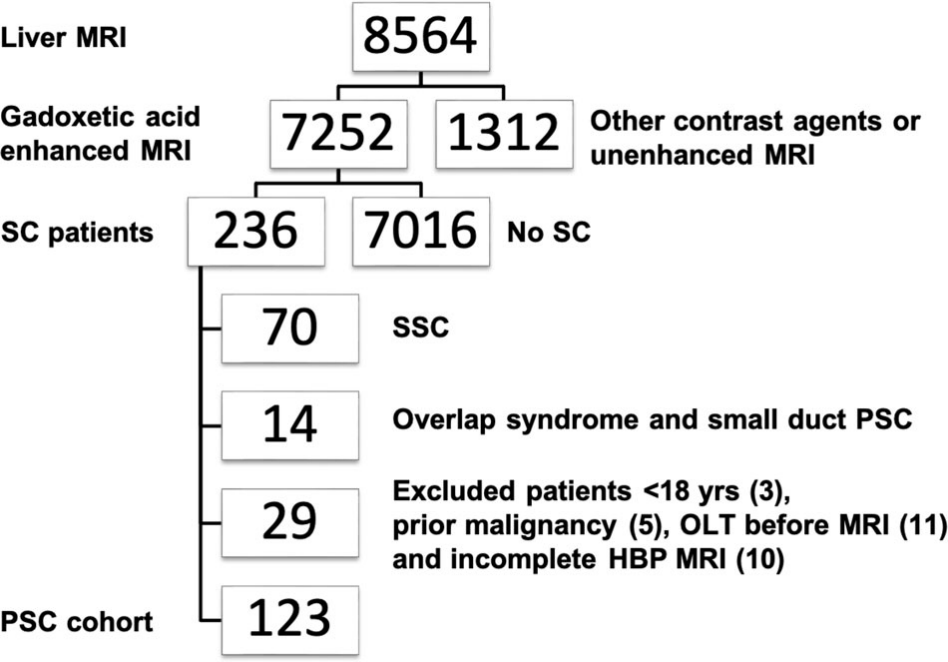
Flowchart Between 2007 and 2022, 8564 patients underwent a standardized 3.0 Tesla contrast-enhanced MRI of the liver. Of these, 1312 patients were excluded because they were imaged using a contrast agent other than gadoxetic acid. A diagnosis other than sclerosing cholangitis further eliminated 7016 patients. Among patients with sclerosing cholangitis, additional exclusion occurred due to: secondary sclerosing cholangitis (SSC) (70), either overlap syndrome and/or small-duct PSC (14), incomplete HBP (10), previous OLT (11), age under 18 years (3), and prior malignancy (5)

Our institutional ethics review board approved this retrospective, single-center study. All patients gave written, informed consent for MRI and interventional procedures. Only patients with confirmed PSC according to EASL guidelines [11] who underwent GA-MRI between October 2007 and March 2022 were included. Patients with small-duct PSC, secondary sclerosing cholangitis, confounding liver illnesses (autoimmune hepatitis (AIH), Primary biliary cholangitis (PBC), Alcoholic liver disease (ALD), etc.), current or prior malignancy, who underwent orthotopic liver transplantation (OLT) prior to MRI, were under the age of 18, and/or had incomplete GA-MRI exams were excluded. Only patients with PSC-associated malignancies, i.e., cholangiocarcinoma, hepatocellular carcinoma (HCC), and gallbladder cancer were enrolled in the study. Patients with current or prior malignancies unrelated to PSC e.g., lung cancer, etc., were also excluded. Thus, our final cohort was comprised of adults with large-duct PSC who had at least one multiparametric GA-MRI, including conventional 2D- and 3D-T2-weighted-MRCP (Fig. 1 Flowchart).

### Clinical data

Demographic and clinical data obtained from electronic medical records included patient age, gender, body mass index (BMI), date of, and indication for MRI, follow-up imaging exams, duration of PSC, and the presence of liver cirrhosis or concomitant inflammatory bowel disease (Table 1). Laboratory tests performed within two weeks of MRI, plus clinical scores that indicated disease severity, including MELD [12], Revised Mayo-Risk-Score (RMRS) [4], Fib-4 [13], APRI [14], ALBI [15], UK-PSC risk scores [16] and Prognostic Index of the Amsterdam-Oxford model (PI-AOM) [17], were recorded (Table 2). Clinical events that occurred after inclusion, orthotopic liver transplantation (OLT), death, cause of death, and cirrhosis decompensation defined as the occurrence of variceal bleeding, ascites, hepatic encephalopathy, or hepatorenal syndrome were recorded.

**Table 1:** Characteristics of 123 primary sclerosing cholangitis (PSC) patients

Main Characteristics	Total number of patients *n* = 123	No sequelae *n* = 86	Sequelae *n* = 37	*p* value
Mean Age (years)	40.5 ± 14.2	38.7 ± 13.7	46.9 ± 14.4	0.001
Female	50 (43.7%)	41	9	0.381
Male	73 (56.3%)	45	28	
Mean BMI (kg/m²)	23.6 ± 4.4	23.8 ± 4.5	22.6 ± 3.9	0.222
Years of disease	7.4 ± 6.2	6.2 ± 5.6	9.3 ± 7.1	0.038
Age at PSC diagnosis (years)	36.3 ± 14.3	35.2 ± 14.4	40.4 ± 13.5	0.066
Time from PSC diagnosis to MRI (years)	4.6 ± 6.5	3.9 ± 6.2	6.9 ± 7.4	0.024
Follow-up time from MRI (years)	3.9 ± 2.7	4.2 ± 2.8	3.3 ± 2.5	0.067
Disease extent at diagnosis				
Intrahepatic only	92 (74.8%)	77 (83.7%)	15 (16.3%)	0.010
Intrahepatic + extrahepatic	31 (25.2%)	9 (29.0%)	22 (71.0%)	
Extrahepatic only	0	0	0	
Liver related Death	7 (5.6%)	0	7 (100%)	0.009
OLT	17 (19.5%)	0	17 (100%)	< 0.0001
Ascites	24 (19.5%)	0	24 (100%)	0.0004
Encephalopathy	7 (5.7%)	0	7 (100%)	0.010
Variceal bleeding	2 (1.6%)	0	2 (100%)	0.047
Decompensation only total	13 (10.6%)	0	13100%)	< 0.0001
Hepatobiliary cancers total	6 (4.9%)	4 (66.7%)	2 (33.3%)	0.021
CCA	4 (3.3%)	2 (50%)	2 (50%)	0.021
Gallbladder cancer	2 (1.6%)	1 (50%)	1 (50%)	n/a
HCC	0	0	0	n/a
Inflammatory Bowel Disease	81 (65.0%)	62 (76.2%)	19 (23.8%)	0.511
Ulcerative colitis	49 (39.8%)	40 (81.6%)	9 (18.4%)	0.435
Crohn’s disease	32 (26.0%)	22 (68.8%)	10 (31.2%)	0.14
Ursodiol	123 (100%)	86 (70.0%)	37 (30.0%)	n/a
Fatty Liver disease	1 (0.8%)	1 (100%)	0	1.000
Cholecystectomy	3 (2.4%)	2 (66.7%)	1 (33.3%)	0.528
Arterial Hypertension	8 (6.5%)	5 (62.5%)	3 (37.5%)	0.372
Diabetes	1 (0.8%)	1 (100%)	0	1.000

Data presented for the means ± standard deviation or absolute numbers and percentage of the group.

*BMI* body mass index, *CCA* cholangiocarcinoma, *HCC* hepatocellular carcinoma, *OLT* orthotopic liver transplant.

* Although 37 patients met an endpoint, 3 of them received an OLT for symptoms of PSC-related cholangitis, not end-stage liver cirrhosis.

**Table 2:** Clinical scores, splenic volume, laboratory tests in 123 patients

Score/Parameter	All patients *n* = 123	No adverse outcome	Adverse outcome	*p* value	Normal Range
MELD	14.02 ± 7.89	10.20 ± 2.84	22.57 ± 8.86	**< 0.001**	6–40
Revised Mayo Risk Score	−0.16 ± 1.35	−0.70 ± 0.87	0.97 ± 1.41	**< 0.001**	
FIB4 index	1.88 ± 2.57	1.45 ± 1.65	3.34 ± 3.35	**< 0.001**	< 1.45
APRI	0.91 ± 1.71	0.69 ± 1.81	1.42 ± 1.49	**0.011**	< 0.5
ALBI	−2.76 ± 0.68	−3.01 ± 0.46	−2.24 ± 0.74	**< 0.001**	≤ −2.60
Short‐Term UK‐PSC Risk Score	−3.06 ± 0.89	−3.43 ± 0.46	−2.32 ± 0.99	**< 0.001**	
Amsterdam-Oxford Model for PSC	1.66 ± 0.89	1.33 ± 0.66	2.30 ± 0.93	**< 0.001**	
Spleen volume (cm³)	408 ± 263	321.97 ± 161.7	586.92 ± 336.55	**< 0.001**	^a^107–315 cm³
Thrombocytes (10^9^/L)	265.7 ± 131.1	288.73 ± 112.3	224.84 ± 156.79	**0.027**	150,000–450,000
Albumin (g/L)	41.71 ± 5.98	43.45 ± 4.72	37.83 ± 6.33	**< 0.001**	35–52
Bilirubin (mg/dL)	2.02 ± 4.43	0.99 ± 1.81	4.23 ± 7.18	**< 0.001**	0.1 to 1.2
Creatinine (mg/dL)	0.92 ± 0.93	0.94 ± 1.08	0.88 ± 0.76	0.334	0.7 to 1.3
GGT (U/L)	246.14 ± 330.9	247.84 ± 823	259.95 ± 352.88	**< 0.001**	< 55
GOT/AST (U/L)	63.94 ± 64.92	56.14 ± 64.69	84.53 ± 66.03	**0.030**	< 36
GPT/ALT (U/L)	80.84 ± 121.70	81.82 ± 141.45	85.05 ± 73.69	0.434	< 45
AP (U/L)	249.4 ± 232.3	218.16 ± 173.9	227.40 ± 320.95	**0.044**	40–129
CRP (mg/L)	1.21 ± 2.01	0.89 ± 1.26	1.56 ± 2.31	**0.047**	< 0.3
Fibrinogen (mg/dL)	396 ± 107.3	393.88 ± 97.32	364.89 ± 126.47	0.575	200–400
CA19-9 (U/mL)	29.6 ± 2089.1	29.82 ± 52.31	82.89 ± 36	0.113	0–37
CEA (ng/mL)	2.34 ± 2.70	2.17 ± 2.47	2.39 ± 2.25	0.680	0–2.5
AFP (ng/mL)	2.46 ± 1.98	2.44 ± 1.61	1.79 ± 1.20	0.405	0–40

Bold values indicate statistical significance *p* < 0.05

Values are expressed as mean ± standard deviation or median (interquartile range)

Student’s *t*-test (Welch corrected in case of unequal variances) or Wilcoxon Rank-Sum test

*MELD* model for end-stage liver disease, *FIB 4* Fibrosis-4 index for liver fibrosis, *APRI* AST-to-platelet ratio index, *ALBI* Albumin-Bilirubin, *GGT* Gamma-glutamyl transferase, *GOT* Glutamic oxaloacetic transaminase, *GPT* Glutamate PyruvateTransaminase, *AP* Alkaline phosphatase, *CRP* C-reactive protein, *CA 19-9* carbohydrate antigen 19-9, *CEA* carcinoembryonic antigen, *AFP* alpha-fetoprotein, *AST* serum aspartate aminotransferase level

MELD = 9.57 × ln(creatinine [mg/dL]) + 3.78 × ln(bilirubin [mg/dL]) + 11.20 × ln(INR) + 6.431 [12]

Revised Mayo Risk Score = 0.0295 × age [years] + 0.5373 × ln(total bilirubin [mg/dL]) − 0.8389 × (serum albumin [g/dL]) + 0.5380 × ln(AST [IU/L]) + 1.2426 × (points for variceal bleeding)

Points for variceal bleeding: 0 if none, 1 if present [4]

FIB-4 = Age [years] × AST Level [U/L] / Platelet Count [10^9^/L] × √ALT [U/L] [13]

APRI score = (AST/upper limit of normal)/platelet count [10^9^/L]) × 100 [14]

ALBI score = −0.085 × (albumin [g/L]) + 0.66 × log (bilirubin [μmol/L]), where: log indicates the logarithm to the base of 10 [15]

Short-term UK-PSC Risk Score = 0.745 × (Bili_Group 1 [0/1]) + 1.613 × (Bili_Group 2 [0/1]) – 0.061 × albumin [g/l] – 0.012 × Hb [g/l] – 0.476 × (Platelet_Group 1 [0/1]) – 0.698 × (Platelet_Group 2 [0/1]) – 0.962 × (Platelet_Group 3 [0/1])

Bili_Group 1 [0/1] … substitute with 1 if bilirubin is between 35 and 50 mg/dL, otherwise 0

Bili_Group 2 [0/1] … substitute with 1 if bilirubin is over 50 mg/dL, otherwise 0

Platelet_Group 1 … substitute with 1 if platelet count is between 150 and 200 10^9^/L, otherwise 0

Platelet_Group 2 … substitute with 1 if platelet count is between 200 and 400 10^9^/L, otherwise 0

Platelet_Group 3 … substitute with 1 if platelet count is over 400 10^9^/L, otherwise 0 [16]

Raw PI-AOM score = 0.323 × PSC_subtype + 0.018 × Age at diagnosis [years] – 2.485 × log_10_(albumin [xLLN]) + 2.451 × abs (log_10_(platelet count [xLLN])-0.5) + 0.347 × log_10_ (AST[xULN]) + 0.393 × log_10_(ALP [xULN]) + 0.337 × log_10_(bilirubin[xULN]). PSC_subtype … substitute with 1 if large duct PSC, substitute with 0 if small duct PSC [17]

^a^[28]

### Definition of sequelae

Patients entered the survival analyses at the time of GA-MRI. In March 2022, patient records were censored at date last seen, if they did not experience any sequelae. Survival status (alive, deceased), and date and type of whichever liver-related event first occurred were recorded (Table 1). OLT, death and decompensation (including encephalopathy, ascites, bleeding of oesophageal varices) were recorded as sequelae. The new occurrence of cholangiocarcinoma (CCA), gallbladder cancer, or hepatocellular carcinoma (HCC) were noted but not considered as sequelae if patients were still alive at the end of the study. Diagnostic or therapeutic endoscopic retrograde cholangiopancraticography (ERCP) was not considered as an event.

### Disease severity classification

PSC severity and expected prognosis were based upon previously validated scores, including the RMRS, Fib-4, APRI, ALBI, Short-Term UK-PSC risk score, and PI-AOM for PSC [7, 16, 18, 19] (Table 2). We further classified these continuous scores into categorical groups, e.g., RMRS: low-risk (≤ 0); intermediate-risk (> 0 and < 2); and high-risk (≥ 2) groups [20, 21]. Binary classification of the Fib-4 was 0 (≤ 1.3) and 1 (> 1.3) [22], and the APRI was 0 (≤ 1.17) and 1 (> 1.17) [22]. The ALBI score was grouped into ≤ −2.60 (Grade 1), >  −2.60 to ≤ −1.39 (Grade 2), and > −1.39 (Grade 3) [23, 24], and the PI-AOM into low-risk, low – intermediate-risk, moderate-risk, and high-risk [25] (Table 2).

### MRI exam protocol

All examinations were performed on a 3 Tesla MR (MAGNETOM Trio Tim, or PrismaFit, Siemens). T2-weighted-MRCP was performed according to Hoeffel et al’s protocol and adhered to International PSC Study Group recommendations [26, 27]. MRCP images included a respiratory-triggered, 3D, heavily T2-weighted sequence in the coronal plane and a breath-hold, thick slab, single-shot, 2D, heavily T2-weighted sequence in the coronal and oblique coronal projections. Dynamic MR images were obtained in the, following transverse plane covering the whole liver during end-expiratory breath hold. T1-weighted VIBE images were obtained pre-contrast and during arterial (using automatic bolus tracking, using TWIST sequence (TR: 68.77, TE: 1.52, FA: 30°, slice thickness: 20 mm, FOV: 350, matrix 148 × 192, and only one NEX), portal venous (70 s), transitional (300 s) and hepatobiliary (20 min) phases (HBP) after injection of gadoxetic acid (Primovist® in Europe, Eovist® in USA). In addition, an axial T2-weighted sequence, an axial T2- weighted sequence with fat-suppression, axial in- and out-of-phase T1-weighted gradient echo sequence and diffusion-weighted sequences with three *b*-values (50, 300 and 600) and ADC map, and axial and coronal plane HBP images (with a flip angle of 20° and 35°) were obtained. The examination parameters for the whole MRI exam are given in Table 1S.

### Image analysis

MRI exams were anonymized, then independently evaluated on a commercially available PACS workstation by five readers, R-A, R-B, R-C, R-D and R-E with 2, 3, 4, 6 and > 20 years of experience in abdominal radiology, respectively. To assess the intra-reader agreement, R-C and R-E reviewed the images twice, at least 12 weeks apart.

Blinded to all clinical data except PSC diagnosis, the radiologists assessed the ANALI scores for each patient. The individual parameter for Anali scores, including intrahepatic bile duct dilatation (IHBD) was assigned as = 0 if any duct was 3 mm or smaller, = 1 if any duct was 4 mm, and = 2 if ducts was 5 mm or larger. Liver dysmorphia was considered present, score = 1, if there was atrophy, lobulation of the liver contour and/or increased caudate-to-right liver lobe ratio [6, 7]. Otherwise, dysmorphia was considered absent, i.e., score = 0. Portal hypertension (PH) was considered present if there were collateral vessels, with or without splenomegaly, score = 1, were observed. Otherwise, PH was scored = 0, indicating its absence. Liver enhancement was assessed on both the arterial (AP)- and hepatobiliary-phase images (HBP). If parenchymal enhancement was uniform, it was scored = 0. Otherwise, heterogeneous liver parenchymal enhancement was scored = 1.

Then Anali scores were calculated as follows: *ANALI*_*NoGd*_ = (1 × dilatation of intrahepatic bile duct (IHBD)) + (2 × liver deformity) + (1 × PH), range 0 to 5, and *ANALI*_*Gd*_ = (1 × liver deformity) + (1 × parenchymal enhancement heterogeneity), range 0 to 2 [6, 7].

Thereafter, Anali scores were dichotimized as follows, ANALI_NoGd_: low risk (0–2 points) and high risk (3–5 points), i.e., binary, ≤ 2 and > 2. ANALI_Gd_ in AP and HBP: low risk and high risk, i.e., binary ≤ 1 and > 1.

On 20-minute HBP images, patients were also dichotomized into normal contrast excretion through the biliary system at 20 min (NFS) or impaired excretion (PFS), i.e., no contrast seen either to: first-order left hepatic duct (LHD) or right hepatic duct (RHD) or common hepatic duct (CHD/hilum) or common bile duct (CBD) or none at all at 20 minutes [10]. For further details regarding PFS, please refer to Poetter-Lang et al [10].

Furthermore, estimated splenic volume was calculated as [mL] = 30 + 0.58 × L × D × T [28]. A cut-off value of 381.1 cm³ was chosen to differentiate normal-sized from enlarged spleens (Table 2) [29].

### Statistical analysis

Metric data are presented as means ± standard deviations or median and quartile, depending upon their distribution. Nominal data are presented as absolute frequencies and percentages. Categorical data were evaluated by the chi-squared-test or the Fisher’s exact test.

Inter- and intrareader agreement between radiologists were assessed using Fleiss,’ and Cohen’s kappa, respectively. The Fleiss’ kappa was determined separately for each parameter, as well as low-risk vs high-risk (i.e., binary) for all 3 ANALI scores, indicating inter-reader agreement. For reader E, the intra-reader Cohen’s kappa was obtained. Then 95% confidence intervals (CI) were calculated for each value.

The kappa values < 0 indicated poor, 0.00–0.20 slight, 0.21–0.40 fair, 0.41–0.60 moderate, 0.61–0.80 substantial, and 0.81–1.00 almost perfect agreement [30]. Event-free survival was defined as the time interval from MR diagnosis to the first liver-related event occurrence. Kaplan-Meier estimates were performed, survival curves compared with log-rank test, when applicable. Univariate Cox proportional hazards regression analysis was performed to evaluate the association between outcomes and binary data for all 4 imaging parameters, i.e., PFS vs NFS, and (low-risk vs high-risk) ANALI scores, clinical scores and laboratory parameters. Multivariate analysis with adjustment for age and sex was also performed.

For event-free survival analysis, we calculated the results of each reader separately and then averaged them, yielding a mean event-free survival for the PFS and each ANALI score.

For survival analyses, cohort data were dichotomized into low- and high-risk scores, for ANALI_NoGd_, i.e., ≤ 2 and > 2, and for both the ANALI_Gd_AP and ANALI_Gd_HBP, i.e., ≤ 1 and > 1. The level for statistical significance was set at *p* < 0.05. Statistical analyses were performed using R Studio (Version 1.4.1717) and IBM SPSS (version 26).

## Results

### Cohort characteristics

We included 123 patients, 50 F/73 M, mean age 40.5 ± 14.2 years (range, 18.3–77.6 years), diagnosed with PSC according to society-guideline MRCP features [27, 31]. Of these, 44 (34.1%) had biopsy-confirmed PSC. Inflammatory bowel disease was histologically verified in 81 patients: 49 with ulcerative colitis; and 32 with Crohn’s disease. Mean duration of the PSC was 7.4 years. Mean follow-up post-MRI was 3.9 years (Table 1).

By March 1st, 2022, 37 (30%) of our 123 patients had experienced at least one sequelae [17 OLT (14%), 7 liver- related deaths (6%), and 13 only pure decompensation (11%)]. The causes of death were hepatic dysfunction or acute-on -chronic liver failure (ACLF) in 4 patients, CCA in 1 patient, gallbladder carcinoma in 1 patient and septic shock of biliary origin in 1 patient. The reasons for OLT were end-stage liver disease with hepatic dysfunction in 14 patients and recurrent cholangitis in 3 patients.

Other recorded events included development of hepatobiliary cancers, i.e., an additional 3 CCA and 1 gallbladder cancer, but no HCCs. These 4 cancers were not considered sequelae since patients were still alive at the end of the study, in accordance with the original study design [7].

There was no statistically significant difference in gender or BMI between both groups (all *p* > 0.05) However, patients with sequelae were significantly older, *p* = 0.001 and their PSC duration was almost 1.5 times longer, *p* = 0.038 (Table 1).

### Inter- and intrareader agreement

Inter-reader agreement for PFS vs NFS, for each variable of the three ANALI scores with and without gadoxetic acid, and for the binary, i.e., low-risk vs high-risk of the three ANALI scores with and without gadoxetic acid are shown in Table 3. All components and scores demonstrated statistical significance, *p* < 0.001. Fleiss’ kappa (ϰ) was highest, 0.81 for binary PFS showing almost perfect agreement between the five readers. Individual variables of the ANALI scores exhibited moderate to substantial agreement (0.41–0.74). Intrahepatic duct dilatation and arterial-phase parenchymal enhancement heterogeneity had the lowest agreement (for both ϰ = 0.41, moderate). Liver deformity had higher agreement (ϰ = 0.74, substantial), while evaluation of portal hypertension and HBP parenchymal enhancement heterogeneity had moderate agreement (ϰ = 0.41 and 46), respectively.

**Table 3:** Inter-reader agreement for all 5 readers using Fleiss’ Kappa correlation with 95% confidence intervals (CI) of potential functional stricture (PFS) and ANALI scores and their components

Parameter	Fleiss’ Kappa (95% CI)	*p* value
Potential functional stricture (PFS) Excretion (yes or no)		
PFS	0.81 (0.75–0.86)	< 0.001
Anali score variables		
IHBD^†^ dilatation	0.41 (0.37–0.45)	< 0.001
Liver deformity	0.74 (0.68–0.79)	< 0.001
Portal hypertension	0.49 (0.43–0.54)	< 0.001
Parenchymal enhancement heterogeneity AP	0.41 (0.35–46)	< 0.001
Parenchymal enhancement heterogeneity HBP	0.46 (0.40–0.51)	< 0.001
ANALI_NoGd_ (0–5)	0.31 (0.28–0.34)	< 0.001
ANALI_Gd_AP (0–2)	0.43 (0.39–0.47)	< 0.001
ANALI_Gd_HBP (0–2)	0.47 (0.43–0.51)	< 0.001
ANALI_NoGd_ binary ≤ 2 and > 2 (low-risk vs high-risk)	0.55 (0.49–0.60)	< 0.001
ANALI_Gd_AP binary ≤ 1 and > 1 (low-risk vs high-risk)	0.53 (0.48–0.59)	< 0.001
ANALI_Gd_HBP binary ≤ 1 and > 1 (low-risk vs high-risk)	0.64 (0.59–0.70)	< 0.001

^†^IHBD, intrahepatic bile ducts

*ANALI*_*NoGd*_ Anali score without gadolinium, *ANALI*_*Gd*_*AP* Anali score with gadoxcetic acid, arterial-phase, ANALI_Gd_HBP Anali score with gadoxcetic acid, hepatobiliary phase

Landies and Koch:

The kappa statistic: Values < 0 as indicating poor and 0.00–0.20 as slight, 0.21–0.40 as fair, 0.41–0.60 as moderate, 0.61–0.80 as substantial, and 0.81–1.00 as almost perfect agreement

The ANALI_NoGd_ showed fair agreement (ϰ = 0.31), versus moderate agreement for the ANALI_Gd_HBP and ANALI_Gd_AP (ϰ = 0.43 and ϰ = 0.47), respectively.

Cohen´s kappa for R-E was 0.87 for the PFS (almost perfect), but only 0.69, 0.72, and 0.77 for ANALI scores, respectively, i.e., substantial to almost perfect, agreement. For R-C. Cohen´s kappa values were 0.82 for PFS (almost perfect), but only 0.65, 0.71, and 0.73 for ANALI scores, respectively i.e., also substantial to almost perfect, agreement.

### Prognostic performance of the imaging and clinical scores

Mean adjusted hazard ratios for low- vs high-risk imaging and clinical scores were as follows: ANALI_NoGd,_ (6.12), PFS, (3.12), ANALI_Gd_AP, (3.59), ANALI_Gd_HBP, (3.56), APRI (3.26), ALBI (3.69), and AP (3.26) (Table 4).

**Table 4:** Mean low- vs high-risk estimates for clinical outcomes (i.e., liver-related death, liver transplantation or decompensated cirrhosis) for PFS and ANALI scores for readers A-E, as well as splenic volume and clinical scores

Parameter	HR^†^ (CI 95%)	*p* value	HRadj^‡^ (CI 95%)	*p* value
Mean PFS vs NFS binary	3.15 (1.63–6.09)	< 0.001	3.12 (1.72–6.41)	< 0.001
Mean ANALI_NoGd_ score binary	6.42 (3.33–12.37)	< 0.001	6.12 (3.22–11.94)	< 0.001
Mean ANALI_Gd_AP score binary	3.79 (1.20–7.19)	< 0.001	3.59 (1.89–6.83)	< 0.001
ANALI_Gd_HBP score binary	3.66 (1.92–6.99)	< 0.001	3.56 (1.86–6.81)	< 0.001
Spleen volume binary	4.08 (2.04–8.16)	< 0.001	4.36 (2.14–8.89)	< 0.001
RMRS binary	4.32 (2.09–8.92)	< 0.001	4.13 (1.93–8.85)	< 0.001
MELD binary	1.11 (1.08–1.15)	< 0.001	1.12 (1.08–1.16)	< 0.001
Fib-4 binary	2.98 (1.55–5.72)	< 0.001	2.99 (1.45–6.15)	< 0.001
APRI binary	3.20 (1.65–6.19)	< 0.001	3.26 (1.68–6.34)	< 0.001
ALBI binary	3.94 (2.01–7.74)	< 0.001	3.69 (1.84–7.38)	< 0.001
AP binary	3.26 (1.36–7.83)	0.008	3.26 (1.36–7.85)	0.008
PI-AOM binary	2.00 (1.40–2.86)	< 0.001	2.01 (1.38–2.92)	< 0.001

Anali score without gadolinium = ANALI_NoGd_, 0–2 = ≤ 2 and 3–5= and > 2 (low-risk vs high-risk), i.e.,

Anali score with gadoxcetic acid, arterial-phase = ANALI_Gd_AP, (0–2 points) (low-risk vs high-risk), i.e., ≤ 1 and > 1

Anali score with gadoxcetic acid, hepatobiliary phase = ANALI_Gd_HBP, ≤ 1 and > 1 (low-risk vs high-risk)

*PFS* potential functional stricture, *NFS* no functional strictures, *HR* hazard ration, *HR adj* adjusted hazard ratio, *CI* confidence interval, *RMRS* revised mayo risk score, *MELD* model endstage liver disease, *APRI* AST-to-platelet ratio index, *ALBI* albumin bilirubin score, *AP* alkaline phosphatase, *PI-AOM* Amsterdam-Oxford model

A cutoff value of 381.1 cm³ was chosen to differentiate normal-sized from enlarged spleens [28]

RMRS: low-risk (≤ 0); intermediate-risk (> 0 and < 2); and high-risk ≥ 2) groups [16, 20]

Fib-4: low-risk (≤ 1.3) and high-risk (> 1.3) [21],

APRI: low-risk (≤ 1.17) and high-risk (> 1.17) [21]

ALBI score: Grade 1 (≤ −2.60), Grade 2 (> −2.60 to ≤ −1.39), and Grade 3 (> −1.39) [22, 23],

PI-AOM: low-risk, low – intermediate-risk, moderate-risk, and high-risk

All mean reader imaging scores (i.e., PFS and 3 ANALI scores) differed significantly (*p* < 0.0001) in patients with vs without sequelae (Table 5).

**Table 5:** Mean of scores for readers A-E per sequelae for the 123 primary sclerosing cholangitis (PSC) patients

MR metric	Risk category	Sequelae		Total	*p value
		0 = no	1 = yes		
Mean Excretion-HBP_MRC	NFS	69 (82.1%/81.2%)	15 (17.9%/39.5%)	84 (68.3%)	< 0.0001
	PFS	16 (41.0%/18.8%)	23 (59.0%/60.5%)	39 (31.7%)	
	Total	85 (69.1%)	38 (30.9%)	123 (100.0%)	
Mean ANALI_NoGd_	Low risk	81 (95.2%/83.5%)	16 (42.1%/%)	97 (78.9%)	< 0.0001
	High risk	4 (4.8%/15.4%)	22 (57.9%/84.6%)	26 (21.1%)	
	Total	85 (69.1%)	38 (30.9%)	123 (100.0%)	
Mean ANALI_Gd_AP	Low risk	76 (89.4%/80.0%)	19 (50.0%/20.0%)	95 (77.2%)	< 0.0001
	High risk	9 (10.6%/32.1%)	19 (50.0%/67.9%)	28 (22.7%)	
	Total	85 (69.1%)	38 (30.9%)	123 (100.0%)	
Mean ANALI_Gd_HBP	Low risk	72 (84.7%/81.8%)	16 (40.5%/18.2%)	88 (71.5%)	< 0.0001
	High risk	13 (15.2%/34.2%)	22 (59.5%/57.9%)	38 (30.9%)	
	Total	85 (69.1%)	38 (30.9%)	123 (100%)	
SV	Low risk	65 (76.5%/83.3%)	13 (34.2%/16.6%)	78 (63.4%)	< 0.0001
	High risk	18 (21.2%/42.9%)	24 (60.0%/57.1%)	42 (34.1%)	
	NA	2	1	3	
	Total	85 (69.1%)	38 (30.9%)	123 (100%)	
RMRS	Low risk	66 (77.6%/86.8%)	10 (26.3%/13.2%)	76 (61.8%)	< 0.0001
	High risk	19 (22.4%40.4/%)	28 (73.9%/59.6%)	47 (38.2%)	
	Total	85 (69.1%)	38 (30.9%)	123 (100%)	

ANALI_NoGd_: low risk (0–2 points) and high risk (3–5 points). ANALI_Gd_ in arterial (AP) and hepatobiliary phase (HBP): low risk (< 2 points) and high risk (2 points). RMRS: low-risk (≤ 0); intermediate-risk (> 0 and < 2); and high-risk (≥ 2) groups [16, 20]. A cut-off value of 381.1 cm³ was chosen to differentiate normal-sized from enlarged spleens

*PFS* potential functional stricture, *NFS* no functional stricture, *SV* splenic volume, *RMRS* revised Mayo risk score

*Pearson’s Chi-squared test used

**Fig. 2:**
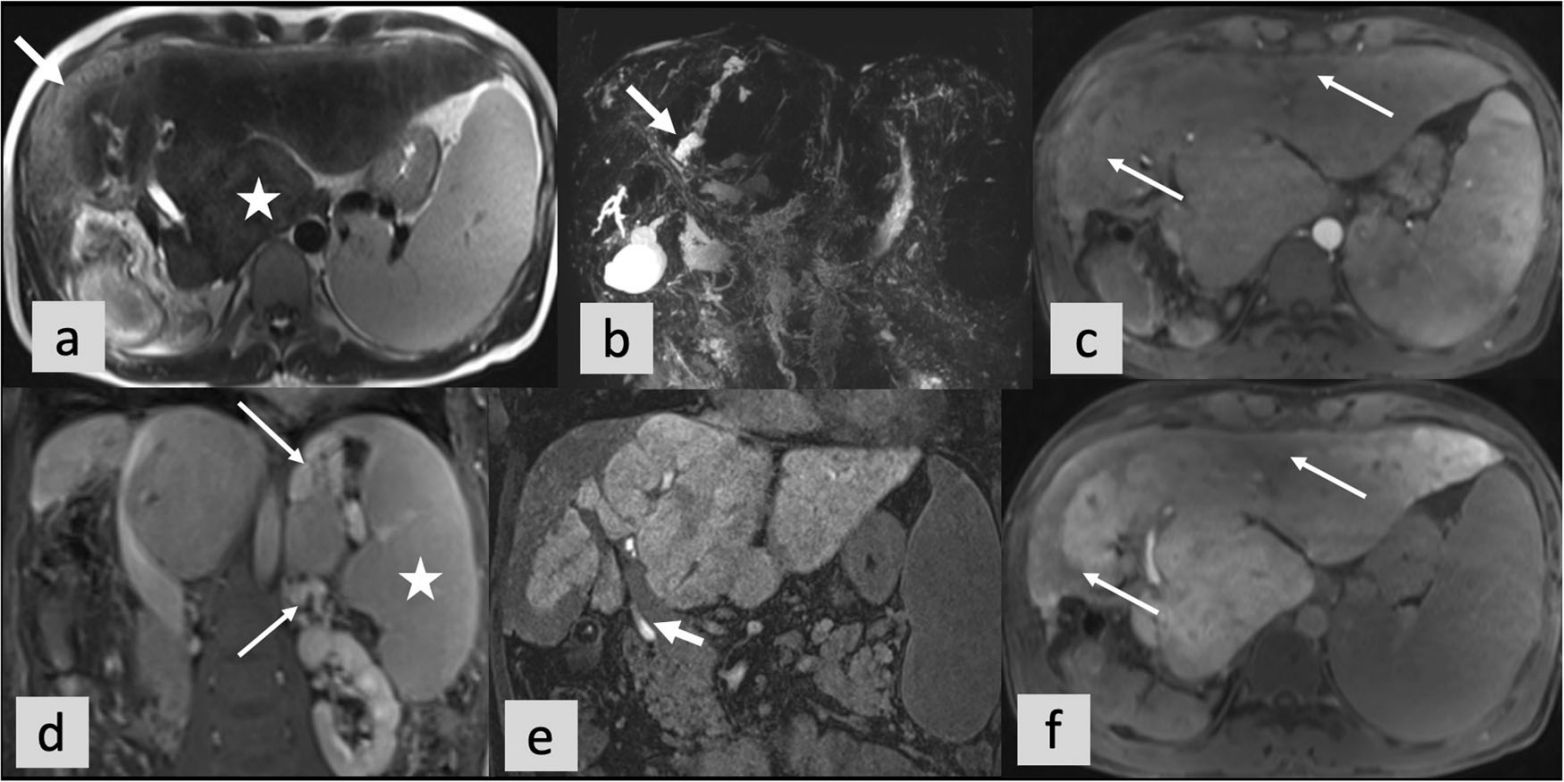
No functional stricture (NFS). OLT for HD. A 36 years-old male PSC patient. Axial (**a**) T2- weighted images, coronal maximum intensity projection from 3D MRCP (**b**) and post-contrast T1- weighted images with fat suppression in arterial (**c**, axial) portal venous phase (**d**, coronal) and HBP (**e**, coronal and **f**, axial). ANALI scores with and without gadoxetic acid in the arterial and HB phases were the same for all five readers (A-E): 2 in the ANALI_Gd_AP, 2 in the ANALI_Gd_HBP, and 5 in the ANALI_NoGd_. In other words, all readers graded duct dilatation ≥ 5 mm, liver dysmorphy (i.e., liver contour lobulation (⇥ in **a**) *±* increased caudate-to-right liver lobe ratio (* in **a**)) as 1, portal hypertension with collateral vessels (⇥ in **d**) *±*, splenomegaly (* in **d**) as 1, and heterogeneous parenchymal enhancement as 1 (⇥ in **c** and **f**). All 5 readers called no functional stricture (NFS) since contrast in the intra- and extrahepatic bile ducts on the HBP image indicates excretion (⇥ in **e**)

The NPV was highest for the ANALI_NoGd,_ 95.2%, while the PPVs for all four scores ranged from 50% to 59.5% (Tables 1, 2 and 3S). See the supplementary section for further data on the mean reader scores and all data on individual scores for all five readers (Table 2S) and (Fig. 2a–c).

**Fig. 3:**
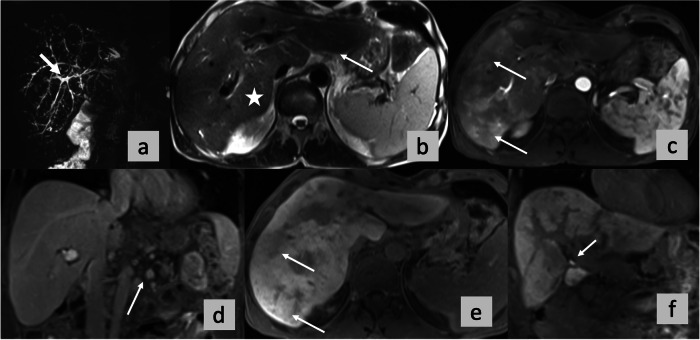
No functional stricture (NFS). OLT for HD. A 55 years-old male PSC patient. Coronal maximum intensity projection from 3D MRCP (**a**), axial T2-weighted image (**b**) and post-contrast T1-weighted images with fat suppression in arterial phase (**c**, axial) and portal venous (**d**, coronal) and HB phase (**e**, axial and **f**, coronal). ANALI_Gd_AP, ANALI_Gd_HBP, and ANALI_NoGd_.scores were calculated by all 5 readers. Readers A-E graded gadoxetic acid-enhanced AP images as 2, and HBP images as 2. In other words, all readers registered parenchymal enhancement heterogeneity as present in the AP (⇥ in **c**), as well as in the HBP (⇥ in **e**). But on non-contrast images, ANALI_NoGd_ scores were 4,4,3,3,2 for readers A, B, C, D, and E, respectively. Reader A, B and D scored the IHBD as 1 (4 mm, ⇥ in **a**), Reader C and E as 0 (≤ 3 mm, ⇥ in **d** in **a**). Reader A, C, and D rated the liver as deformed = 1 (⇥ in **d** in **b**), whereas readers B and E judged the liver as normal = 0. Reader E scored no signs of portal hypertension = 0, whereas, due to collateral vessels (⇥ in **d**), all other readers scored PH as = 1. All 5 readers called no functional stricture (NFS) since contrast excretion is seen within the intra- and extrahepatic bile ducts in the HBP (⇥ in **d**, **f**)

The Kaplan Meier curves for the outcome-free survival of all patients showed a markedly higher probability of sequelae over time in patients with high-risk vs. low-risk imaging scores (Fig. 3a–d). The difference was always most visible one year after MRI, especially for the ANALI_NoGd._ *p* value for log-rank test for all four imaging scores (ANALI_NoGd_, ANALI_Gd_AP, ANALI_Gd_HBP and PFS) was statistically significant (*p* < 0.001).

### Spleen volumetrics

Spleen volumes were significantly higher (*p* < 0.001) in the patients with adverse outcomes and significantly more patients with splenomegaly had liver-related outcomes (*p* < 0.001), highlighting the importance of splenomegaly as a marker of disease severity (Table 2, 3S).

## Discussion

**Fig. 4:**
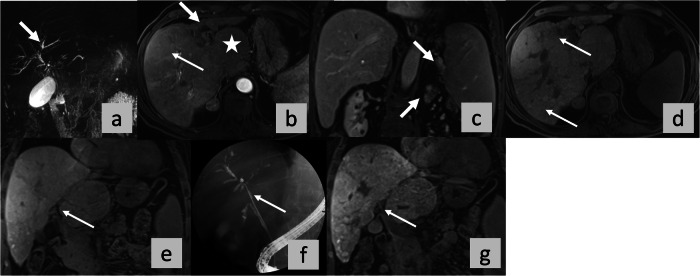
Functional stricture (FS). OLT for HD. A 59 years-old male PSC patient. Coronal maximum intensity projection from 3D MRCP (**a**), Post-contrast T1-weighted images with fat suppression in arterial phase (**b**, axial), portal venous (**c**, coronal) and HB phase (**d**, axial and **e**, coronal). Also, ERCP (**f**, coronal) and 4-week follow-up coronal T1-VIBE HBP, after ERCP (**g**). Potential functional stricture (PFS) was diagnosed by all five readers due to the absent contrast excretion in the HBP ( ↑ in **e**). ERCP the following day confirmed a FS which was dilated (⇥ in **f**). Follow-up HBP shows normal excretion (⇥ in **g**), i.e., NFS, now. All five readers scored the ANALI_Gd_HBP as 2 (⇥ in **d**) because the parenchymal enhancement was rated as heterogeneous and liver dysmorphy was judged present (➞in **b**). However, for the ANALI_Gd_AP, readers A, C and E scored the parenchymal enhancement as heterogeneous (⇥in **b**), i.e., 1 while readers B and D scored the enhancement as homogeneous, i.e., 0. This means the overall ANALI_Gd_AP scores were 2, 1, 2, 1, 2 for readers A, B, C, D, and E, respectively. For the ANALI_NoGd,_ readers A, B, C, D, and E assigned scores of 3,2,5,3, and 2, respectively. For the individual parameters, IHBD were scored as 0, 0,2 [maximal duct diameter as ≥ 3 mm (⇥ in **a**)], 0,0, respectively. All readers felt that liver dysmorphia [enlarged caudate lobe (* in **b**), lobulated liver surface (➞ in **b**)] was present, i.e., 1. All but reader B felt that PH was present due to collateral vessels (⇥ in **c**)

We found that PFS, and all three ANALI scores (ANALI_NoGd,_ ANALI_Gd_AP, ANALI_Gd_HBP) could non-invasively predict PSC outcomes, i.e., liver-related death, the need for OLT, and decompensation. Both the ANALI scores and PFS are derived from a routine gadoxetic acid- enhanced MRI, including T2-weighted MRCP. Although the inter-reader agreement for the raw ANALI scores were only fair to moderate, confirming the results of previous studies [8, 9], this agreement improved substantially when the scores were dichotomized into low- and high-risk. The relatively low inter-reader agreement of the Anali Scores can be explained by tiny differences in reader measurements, quite understandable since we are dealing with millimetres (Fig. 4). Whereas one reader might measure a duct as 3 mm, another might call it 4 mm. By dichotomizing the ANALI scores, we get the overall picture of the patietnt´s disease status, i.e., relatively mild vs relatively severe. In fact, that is the point of prognostication, i.e., to determine the big picture. Furthermore, the predictive value of these binary single- and mean-reader ANALI scores (low- and high-risk) was statistically significant, confirming the value of ANALI scores as an outcome prognosticator [6–9]. On the contrary, the PFS had almost perfect inter- and intra-reader agreement which we attributed to its simplicity, i.e., presence or absence of biliary contrast excretion [10]. Therefore, the combined use of Anali scores and PFS makes it an appealing prognosticator for short and medium-to-long-term-monitoring.

As the PFS and all three ANALI scores correlated well with all clinical scores and laboratory tests in assessing PSC severity, all should be taken into consideration when managing the patient. By considering the whole GA-MRI, one can determine whether ERCP is required, i.e., presence of a PFS, or whether high-risk ANALI scores are due to liver cirrhosis as a consequence of PSC’s natural course, i.e., hepatocellular dysfunction (HD) [6, 7, 10].

A direct relationship has been shown between PFS and sequelae [10], just as has been shown previously for ERCP-diagnosis of dominant stricture (DS) and its sequelae [11, 32, 33]. This highlights the importance of timely diagnosis and treatment of functional stricture (FS) in determining PSC’s course [10]. Like DS, FS may exacerbate cholestasis, leading to further inflammation and fibrosis, and finally, the development of liver cirrhosis and decompensation (HD) requiring OLT [10, 34]. Furthermore, recurrent biliary tract obstruction, i.e., DS or FS, causing recurrent cholangitis may accelerate PSC progression through inflammation and fibrosis [35]. As liver damage is related to stricture severity causing bile flow obstruction [36] PFS is not only a diagnostic tool but is also a predictive surrogate. By making an early diagnosis of functional stricture, prompt ERCP to confirm and treat a dominant stricture may reduce the risk of long-term liver injury in these patients [10, 33].

**Fig. 5:**
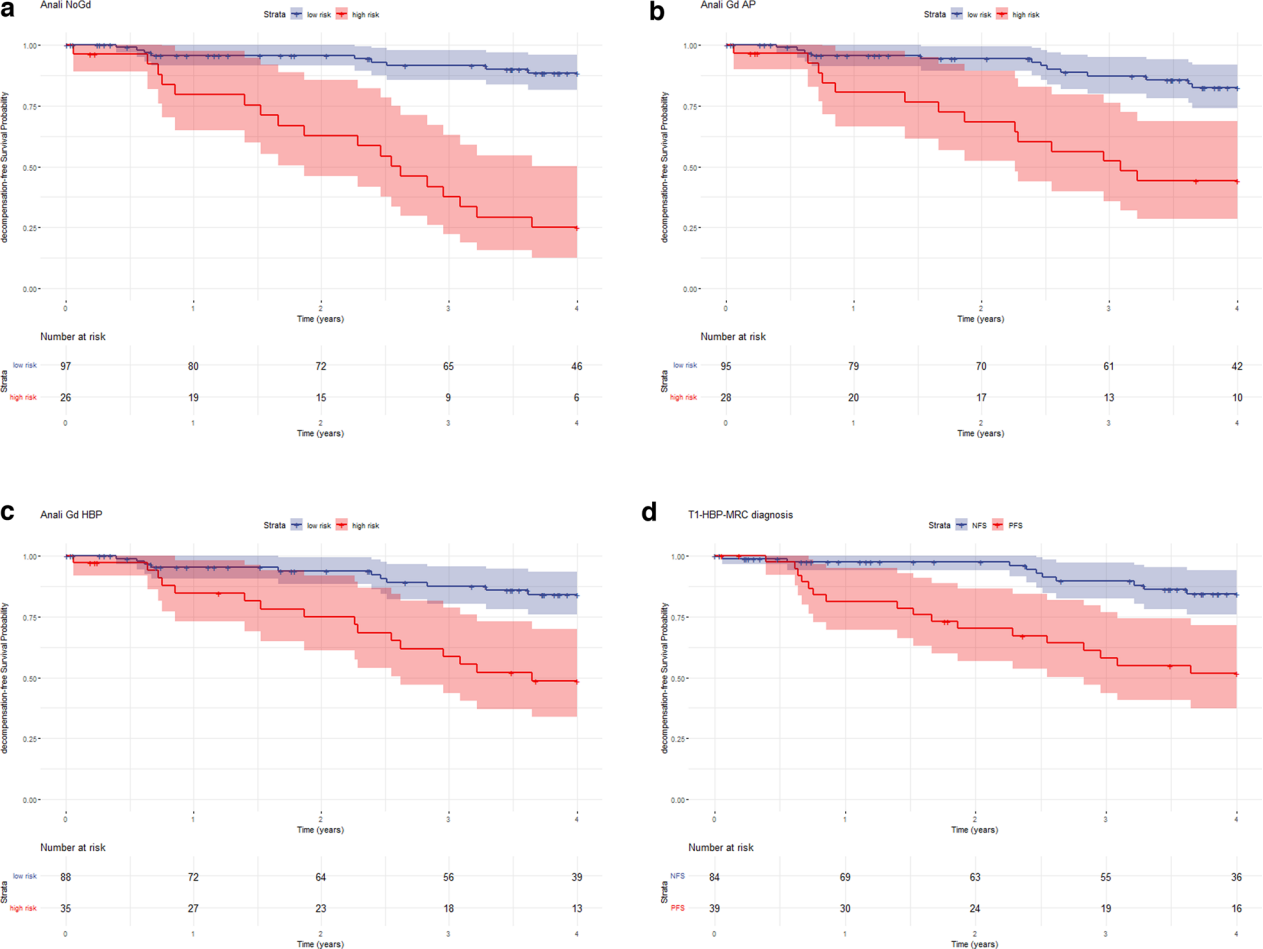
**a**–**d** Kaplan Maier curves for adverse outcome-free survival for the low- vs high-risk PSC patients based upon ANALI_NoGd_, ≤ 2 and > 2 (**a**), ANALI_Gd_AP, ≤ 1 and > 1 (**b**), ANALI_Gd_HBP, ≤ 1 and > 1 (**c**), and PFS vs NFS (**d**)

We observed that ANALI_NoGd_ was a stronger predictor of adverse outcomes than PFS and ANALI_Gd_ (Fig. 5). We attributed this to the fact that it takes into account extrahepatic features of advanced cirrhosis, too, i.e., not just dilatation of intrahepatic bile duct (IHBD) and liver deformity, but also portal hypertension. In addition, the wider range of ANALI_NoGd_, from 0 to 5, rather than 0 to 2 for the ANALI_Gd_, probably stratifies the patients better. However, we have to interpret these results with caution, since the confidence interval (CI) was wide. Further prospective studies are needed.

Even so, the prognostic ability of PFS is comparable to that of both ANALI_Gd_ [6, 7]. This highlights the main advantage of Poetter-Lang et al’s recently-introduced PFS [10]. Because MRI is risk-free compared to invasive ERCP, clinicians likely act far more quickly to work-up suspected FS. The advantage of high inter- and intra-reader agreement with PFS is likely to increase diagnostic confidence, potentially contributing to earlier management of DS if detected. This will probably be just as important for FS as it has been for DS which complicates the clinical course in up to 50% of PSC patients [37]. Thus, imaging can generate reliable prognostic models, and may help avoid or postpone progression to severe fibrosis of end- stage PSC. We suggest that more weight should be given to the PFS if a clinically-relevant stricture is the concern while the ANALI_NoGd_ score should be given preference in long-term counselling.

The mean NPVs were very high (i.e., ca 90%) for all ANALI scores and ca 80% for the PFS, while the PPVs were never more than 50–60%. Such high NPVs can help clinicians identify PSC patients who are unlikely to have an event, i.e., at low risk. This can help to avoid unnecessary invasive tests and treatments.

Furthermore, our results can be implemented to tailor the MR exam according to the clinical indication. That is, for the follow-up of stable PSC patients, we can perform MRI without contrast agent, while in symptomatic PSC patients with elevated liver enzymes or tumor marker (s), we can inject gadoxetic acid. If a PFS is present, ERCP with brush cytology can clarify the presence and nature of the stricture, i.e., either benign or malignant [10]. This should be further evaluated in a prospective multi-center study.

Spleen volume also correlated significantly with PSC adverse outcomes. As in other chronic liver diseases, splenomegaly and imaging signs of portal hypertension herald advanced PSC, and increased risk of further event(s) [29].

Our study had several limitations. First, it was a retrospective single-center study with inherent potential bias. However, we assume that this is not a serious limitation, as this is a conformity study for established scores, i.e., Anali scores. Secondly, the mean follow-up after MRI (3.9 years) was relatively short given the natural course of PSC. Therefore, our results should be interpreted as mid-term prognostic outcomes and further long-term evaluation is warranted. Thirdly, our study population presented for imaging with moderate-to- advanced disease, therefore, the majority of events occurred within 12 months of MRI. As this is not expected in PSC, developing predictive models based on patients with advanced disease might overestimate the risk. We believe this relatively high percentage of adverse events within a short follow-up time is because our tertiary care patients were severely ill compared to the average PSC patient. This is a well-known phenomenon in tertiary centers, such as ours [17]. Furthermore, our retrospective study design, by skewing our inclusion cohort, caused rather short mean follow-up times (3.9 years) as compared to our long observation period, i.e., 7.4 years.

In conclusion, by dichotomizing, the four MR-derived scores, the NPVs, especially for the ANALI_NoGd_, proved good prognosticators. Furthermore, they appear to be complementary, i.e., the ANALI_NoGd_ seems a better longer-term predictor of PSC-related outcomes while the PFS is superior if a potential functional stricture is the main clinical concern.

## Supplementary information

Electronic Supplementary Material

## Abbreviation

ALBI: Albumin-bilirubin score
ANALI_Gd_AP: ANALI score during the arterial phase of GA-MRI
ANALI_Gd_HBP: ANALI score during hepatobiliary phase of GA-MRI
ANALI_NoGd_: ANALI score calculated on non contrast images
AOM: Amsterdam-Oxford model for PSC
APRI: AST to Platelet Ratio Index
BMI: Body mass index
CCA: Cholangiocarcinoma
CI: Confidence Intervals
DS: Dominant stricture
ERCP: Endoscopic retrograde cholangiopancreatography
Fib-4: Fibrosis-4-index
GA-MRI: Gadoxetic acid-enhanced MRI
HBP: Hepatobiliary phase
HCC: Hepatocellular carcinoma
HD: Hepatocellular dysfunction
HR: Hazard ratio
IHBD: Intrahepatic bile duct
MELD: Model for End-Stage Liver Disease
MRI: Magnetic resonance imaging
NFS: No functional stricture
NPV: Negative predictive value
OLT: Orthotopic liver transplantation
PFS: Potential functional stricture
PH: Portal hypertension
PI-AOM: Prognostic Index for AOM
PPV: Positive predictive value
PSC: Primary sclerosing cholangitis
RMRS: Revised Mayo risk score
T2-MRCP: T2-weighted magnetic resonance cholangiopancreatography
