# Supplementary material for: Disease severity prognostication in primary sclerosing cholangitis: a validation of the Anali scores and comparison with the potential functional stricture
Source: Eur Radiol. 2024 Jun 13;34(12):7632–44. doi: 10.1007/s00330-024-10787-4 (PMC11557717; doi:10.1007/s00330-024-10787-4)
Supplement: Supplementary file 1 — Electronic Supplementary Material [file 330_2024_10787_MOESM1_ESM.pdf]

Disease severity prognostication in primary sclerosing cholangitis: a validation of the Anali scores and comparison with the potential functional stricture

Electronic Supplementary Material (ESM)

Eur Radiol (2024) Poetter-Lang S, Ba-Ssalamah A, Messner A et al.

Table 1S. MR protocol with exam parameters

Sequences	Slice orientation	Matrix	Voxel mm	FOV mm	SL mm	Phase Direction	TR ms	TE ms	FA Degree	Time s
T1 VIBE (FLASH 3D) in-phase	Axial	400x320	1.3x1.3x1.7	400	1.7-2	AP	4.4	2.66	20	15
T1 VIBE (FLASH 3D) opposed-phase	Axial	400x320	1.3x1.3x1.7	400	1.7-2	AP	4.4	1.33	20	15
T1 VIBE Dixon unenhanced	Axial	400x320	1.3x1.3x1.7	400	1.7-2	AP	4.4	1.33	20	15
T1 VIBE Dixon gadoxetic-enhanced (arterial and portal-venous phases)	Axial	400x320	1.3x1.3x1.7		1.7-2	AP	4.4	1.33	20	15x3
T1 VIBE Dixon 5 min post contrast (transitional phase)	Axial	400x320	1.3x1.3x1.7		1.7-2	AP	4.4	1.33	20	15
DWI TSE-EPI/ADC	Axial	400x134	1.5x1.5x5	400	5	AP	3400	38	90	314
T2 HASTE fatsat	Axial	400x320	1.3x1.3x5	400	5	AP	1800	154	150	88
T2 HASTE	Coronal	400x256	1.6x1.6x5	400	5	RL	1800	154	143	94
T1 VIBE Dixon 20 min post contrast (HBP)	Axial	400x320	1.3x1.3x1.7	400	1.7-2	AP	4.4	1.33	20<br>35	15
T1 VIBE Dixon 20 min post contrast (HBP)	Coronal	243x320	1.4x1.4x1.5	450	1.5-2	RL	4.5	1.3	20<br>35	18

Footnote. HASTE = Half-Fourier Acquisition Single-shot Turbo spin Echo imaging; DWI TSE-EP/ADC = Diffusion-Weighted Imaging Turbo Spin Echo-Echo-Planar; MRCP= Magnetic Resonance Cholangiopancreatography; MIP=Maximum Intensity Projection; GRE = Gradient echo, VIBE = Volumetric Interpolated Breath-hold Examination, FOV = Field of view, Voxel = Voxel size, SL = Slice thickness, TR = Repetition time, TE = Echo time, FA = Flip angle, Time = Acquisition time.

Table 2S. Risk estimates for sequelae (liver-related death, liver transplantation or hepatic decompensation) of binary categories of PFS and ANALI scores for individual five readers, as well as the Revised Mayo risk score and splenic volumes.

Parameter	HR† (CI 95%)	*P*-value	HRadj‡ (CI 95%)	*P*-value
PFS-Reader A	3.56 (1.91-6.61)	<0.001	3.28 (1.75-6.16)	**<0.001**
PFS-Reader B	3.36 (1.82-6.18)	<0.001	3.05 (1.64-5.67)	**<0.001**
PFS-Reader C	3.84 (2.05-7.19)	<0.001	3.59 (1.91-6.77)	**<0.001**
PFS-Reader D	3.77 (2.01-7.06)	<0.001	3.65 (1.94-6.87)	**<0.001**
PFS-Reader E	3.11 (1.69-5.74)	<0.001	2.87 (1.54-5.34)	**<0.001**
**$ANALI_{NoGd}$** R_A	6.96 (3.71-13.04)	<0.001	6.49 (3.43-12.32)	**<0.001**
**$ANALI_{NoGd}$** R_B	3.96 (2.41-7.32)	<0.001	3.46 (1.94-6.84)	**<0.001**
**$ANALI_{NoGd}$** R_C	7.28 (3.77-14.10)	<0.001	6.72 (3.44-13.14)	**<0.001**
**$ANALI_{NoGd}$** R_D	5.66 (2.96-10.83)	<0.001	5.92 (3.02-11.62)	**<0.001**
**$ANALI_{NoGd}$ R_E**	3.81 (1.97-7.39)	<0.001	3.33 (1.68-6.62)	**<0.001**
**$ANALI_{Gd}$AP** R_A	4.15 (2.18-7.89)	<0.001	3.70 (1.91-7.16)	**<0.001**
**$ANALI_{Gd}$AP** R_B	4.42 (2.39-8.19)	<0.001	3.93 (2.07-7.45)	**<0.001**
**$ANALI_{Gd}$AP** R_C	3.85 (2.04-7.27)	<0.001	3.45 (1.81-6.58)	**<0.001**
**$ANALI_{Gd}$AP** R_D	3.58 (1.93-6.63)	<0.001	3.23 (1.73-6.04)	**<0.001**
**$ANALI_{Gd}$AP R_E**	3.73 (1.98-7.02)	<0.001	3.52 (2.86-6.68)	**<0.001**
**$ANALI_{Gd}$HBP** R_A	4.88 (2.63-9.08)	<0.001	4.52 (2.38-8.57)	**<0.001**
**$ANALI_{Gd}$HBP** R_B	4.09 (2.21-7.56)	<0.001	3.60 (1.87-6.92)	**<0.001**
**$ANALI_{Gd}$HBP** R_C	4.22 (2.24-7.96)	<0.001	3.81 (1.99-7.31)	**<0.001**
**$ANALI_{Gd}$HBP** R_D	4.15 (2.25-7.66)	<0.001	3.72 (1.97-7.02)	**<0.001**
**$ANALI_{Gd}$HBP R_E**	3.72 (1.96-7.08)	<0.001	3.46 (1.80-6.67)	**<0.001**
**SV < 381.1 cm^3 vs ≥ 381.1**	4.03 (2.11-7.33)	<0.001	4.59 (2.34-9.00)	**<0.001**
**RMRS <2 vs ≥ 2**	4.44 (2.27-8.69)	0.001	4.07 (1.99-8.30)	**<0.001**

Anali score without gadolinium= $ANALI_{NoGd}$, Anali score with gadoxcetic acid, arterial-phase= $ANALI_{Gd}$AP, Anali score with gadoxetic acid, hepatobiliary phase= $ANALI_{Gd}$HBP, SV=splenic volume, RMRS=revised Mayo risk score, HR=hazard ratio, HR adj=adjusted hazard ratio, CI=confidence interval

Table 3S. Individual scores for readers A-E per sequelae for the 123 primary sclerosing cholangitis (PSC) patients

MR metric /Reader	Risk category	Adverse Events		Total	*p value
		0	1		
PFS R_A	0	69 (81.2%/ 82.1%)	15 (39.5%/17.9%)	84 (68.3%)	p value: <0.0001
	1	16 (41.0%/18.8%)	23 (59.0%/60.5%)	39 (31.7%)	
	Total	85 (69.1%)	38 (30.9%)	123 (100.0%)	
PFS R_B	0	72 (84.7%/80%)	18 (47.4%/20%)	90 (73.2%)	p value: <0.0001
	1	13 (15.3%/39.4%)	20 (52.6%/60.6%)	33 (26.8%)	
	Total	85 (69.1%)	38 (30.9%)	123 (100.0%)	
PFS R_C	0	68 (80%/82.9%)	14 (36.8%/17.1%)	82 (66.7%)	p value: <0.0001
	1	17 (20%/41.5%)	24 (63.2%/58.5%)	41 (33.3%)	
	Total	85 (69.1%)	38 (30.9%)	123 (100%)	
PFS R_D	0	66 (77.6%/77.6%)	15 (39.5%/22.4%)	81 (%)	p value: <0.0001
	1	19 (22.4%/22.4%)	23 (60.5%/54.8%)	42 (30.9%)	
	Total	85 (69.1%)	38 (30.9%)	123 (100%)	
PFS R_E	0	69 (81.2%/80.2%)	17 (44.7%/19.8%)	86 (69.9%)	p value: <0.0001
	1	16 (18.8%/43.2%)	21 (55.3%/56.8%)	37 (30.1%)	
	Total	85 (69.1%)	38 (30.9%)	123 (100%)	
$ANALI_{NoGd}$ R_A	0	80 (94.1%/83.3%)	16 (42.1%/16.7%)	96 (78%)	p value: <0.0001
	1	5 (5.9%/18.5%)	22 (57.9%/81.5%)	27 (22%)	
	Total	85 (69.1%)	38 (30.9%)	123 (100%)	
$ANALI_{NoGd}$ R_B	0	72 (84.7%/78.3%)	17 (44.7%/21.7%)	89 (72.4%)	p value: <0.0001
	1	13 (15.3%/38.2%)	21 (55.3%/61.8%)	34 (27.6%)	
	Total	85 (69.1%)	38 (30.9%)	123 (100%)	
$ANALI_{NoGd}$ R_C	0	78 (91.8%/83.9%)	15 (39.5%/16.1%)	93 (75.6%)	p value: 0.0020 (Fisher's Exact Test for Count Data)
	1	7 (8.2%/23.3%)	23 (60.5%/76.7%)	30 (24.4%)	
	Total	85	38	123 (100%)	
$ANALI_{NoGd}$ R_D	0	72 (84.7%/85.7%)	12 (31.6%/14.3.%)	84 (68.3%)	p value: 0.0004
	1	13 (15.3%/33.3%)	26 (68.4%/66.7%)	39 (31.7%)	
	Total	85 (69.1%)	38 (30.9%)	123 (100%)	

MR metric /Reader	Risk category	Adverse Events 0	Adverse Events 1	Total	*p value
ANALI$_{NoGd}$ R_E	0	81 (95.2%/75%)	27 (71.1%/25%)	108 (87.8%)	p value: 0.0001
	1	4 (4.8%/26.7%)	11 (28.9%/73.3%)	15 (12.2%)	
	Total	85 (67.4%)	38 (30.9%)	123 (100%)	
ANALI$_{Gd}$AP R_A	0	78 (91.8%/77.2%)	23 (60.5%/22.8%)	101 (82.1%)	p value: <0.0001
	1	7 (8.2%/31.8%)	15 (39.5%/68.2%)	22 (17.9%)	
	Total	85 (67.4%)	38 (30.9%)	123 (100%)	
ANALI$_{Gd}$AP R_B	0	77 (90.6%/78.6%)	21(55.3%/21.4%)	98 (79.7%)	p value: <0.0001
	1	8 (9.4%/32%)	17 (44.7%/68%)	25 (20.3%)	
	Total	85 (67.4%)	38 (30.9%)	123 (100%)	
ANALI$_{Gd}$AP R_C	0	74 (87.1%/72.5%)	21(55.3%/27.5%)	102 (82.9%)	p value: <0.0001
	1	11 (12.9%/37%)	17 (44.7%/63%)	27 (17.1%)	
	Total	85 (67.4%)	38 (30.9%)	123 (100%)	
ANALI$_{Gd}$AP R_D	0	76 (89.4%/78.4%)	21 (55.3%/21.6%)	97 (78.9%)	p value: <0.0001
	1	9 (10.6%/34.6%)	17(44.7%/65.4%)	26(21.1%)	
	Total	85 (68.3%)	38 (30.9%)	123 (100%)	
ANALI$_{Gd}$AP R_E	0	65(76.5%/80.2%)	16 (42.1%/19.8%)	81 (65.9%)	p value: <0.0001
	1	20 (23.5%/47.6%)	22 (57.9%/52.4%)	42 (34.1%)	
	Total	85 (67.4%)	38 (30.9%)	123 (100%)	
ANALI$_{Gd}$HBP R_A	0	75 (88.2%/81.5%)	17(44.7%/19.5%)	92(74.8%)	p value: <0.0001
	1	10 (11.8%/32.3%)	21(55.3%/67.7%)	31 (25.2%)	
	Total	85 (69.1%)	38 (30.9%)	123 (100%)	
ANALI$_{Gd}$HBP R_B	0	76 (89.4%/78.4%)	21(55.3%/21.6%)	97(78.9%)	p value: <0.0001
	1	9 (10.6%/34.6%)	17 (44.7%/65.4%)	26 (21.1%)	
	Total	85 (69.1%)	38 (30.9%)	123 (100%)	
ANALI$_{Gd}$HBP R_C	0	72 (84.7%/80%)	18 (47.4%/20%)	90 (73.2%)	p value: <0.0001
	1	13 (15.3%/39.4%)	20 (52.6%/60.6%)	33 (26.8%)	
	Total	85 (69.1%)	38 (30.9%)	123 (100%)	
ANALI$_{Gd}$HBP R_D	0	74 (87.1%/80.4%)	18 (47.4%/19.6%)	92 (80.4%)	p value: <0.0001
	1	11 (12.9%/35.5%)	20 (52.6%/64.5%)	31 (19.6%)	
	Total	85 (69.1%)	38 (30.9%)	123 (100%)	
ANALI$_{Gd}$HBP R_E	0	63 (74.1%/80.8%)	15 (39.5%/19.2%)	78 (63.4%)	p value: <0.0001
	1	22 (25.9%/48.9%)	23 (60.5%/51.1%)	45 (36.6%)	
	Total	85 (69.1%)	38 (30.9%)	123 (100%)	

Eur Radiol (2024) Poetter-Lang S, Ba-Ssalamah A, Messner A et al.

MR metric /Reader	Risk category	Adverse Events		Total	*p value
		0	1		
SV	0	65 (82.7%/78.8%)	13(17.3%/34.1%)	81 (64.3%)	p value: <0.0001
	1	18(40.0%/21.2%)	24(60.0%/65.9%)	45 (35.7%)	
	NA	2	1	3	
	Total	85 (69.1%)	38 (30.9%)	123 (100%)	
RMRS	0	66 (77.6%/86.8%)	10 (26.3%/13.2%)	76 (61.8%)	p value: <0.0001
	1	19 (22.4%/40.4%)	28 (73.7%/59.6%)	47 (38.2%)	
	Total	85 (69.1%)	38 (30.9%)	123 (100%)	

0=no sequelae, 1=at least one sequelae
Anali score without gadolinium=ANALI$_{NoGd}$, Anali score with gadoxcetic acid, arterial-phase=ANALI$_{Gd}$AP, Anali score with gadoxcetic acid, hepatobiliary phase=ANALI$_{Gd}$HBP, PFS=potential functional strictures, SV=splenic volume, RMRS=revised Mayo risk score

*Pearson’s Chi-squared test used
